# Generation of an infectious clone of HuN4-F112, an attenuated live vaccine strain of porcine reproductive and respiratory syndrome virus

**DOI:** 10.1186/1743-422X-8-410

**Published:** 2011-08-19

**Authors:** Shanrui Zhang, Yanjun Zhou, Yifeng Jiang, Guoxin Li, Liping Yan, Hai Yu, Guangzhi Tong

**Affiliations:** 1 Division of Swine Infectious Diseases, Shanghai Veterinary Research Institute, Chinese Academy of Agricultural Sciences, Shanghai 200241, China

**Keywords:** Infectious clone, PRRSV, HuN4-F112

## Abstract

**Background:**

Nowadays, PRRS has become one of the most economically important infectious diseases of pig worldwide. To better characterize and understand the molecular basis of PRRSV virulence determinants, it would be important to develop the infectious cDNA clones. In this regard, HuN4-F112, a live-attenuated North-American-type PRRSV vaccine strain, could serve as an excellent model.

**Results:**

In the study, genomic sequence of HuN4-F112, an attenuated vaccine virus derived from the highly pathogenic porcine reproductive and respiratory syndrome virus (PRRSV) HuN4 strain, was determined and its full-length cDNA was cloned. Capped RNA was transcribed in vitro from the cDNA clone and transfected into BHK-21 cells. The supernatant from transfected monolayers were serially passaged in Marc-145 cells. The rescued virus exhibited a similar growth pattern to its parental virus in Marc-145 cells with peak titers at 48 h post-infection.

**Conclusion:**

In conclusion, we rescued virus from an infectious cDNA clone of attenuated vaccine. It is possible in the future that a new attenuated PRRSV vaccine with broader specificity and good immunogenicity can be designed in vitro via an infectious cDNA clone platform coupled with validated information on virulence determinants.

## Background

Porcine reproductive and respiratory syndrome (PRRS) was first reported in late 1980s in North America and shortly thereafter in Europe. The disease is characterized by reproductive failure in late gestation in sows and respiratory symptoms in pigs of all ages [1-3]. Nowadays, PRRS has become one of the most economically important infectious diseases of pig worldwide [4,5]. The PRRS virus (PRRSV) is a small enveloped positive-strand RNA virus belonging to the family *Arteriviridae *in the order *Nidovirales *together with equine arteritis virus (EAV), simian hemorrhagic fever virus, and lactate dehydrogenase-elevating virus (LDV) of mice[6,7]. PRRSV is further classified into two distinct genotypes, the North American type and the European type [8,9].

Since May 2006, an atypical PRRS (also known as porcine high fever syndrome) has been pandemic in China. Several studies have confirmed that the causative agent of the outbreaks was highly pathogenic PRRSV (HP-PRRSV) and several HP-PRRSV strains were isolated [10-12]. The genome sequences of several strains representing PRRSV have been determined and infectious cDNA clones were developed [13,14]. However, few of these came from a strain with attenuated virulence. To better characterize and understand the molecular basis of PRRSV virulence determinants, it would be important to develop infectious clone of a virulence-attenuated strain. In this regard, HuN4-F112, a live-attenuated North-American-type PRRSV vaccine strain, could serve as an excellent model.

The present article describes the genomic sequence of a virulence-attenuated PRRSV strain HuN4-F112 and the successful rescuing of the virus from its infectious full-length cDNA clone.

## Materials and methods

### Virus strain and cell line

HuN4-F112 is a virulence-attenuated North-American-type PRRSV strain derived via consecutive passage in Marc-145 cells. This virus strain was maintained in our laboratory as a live-attenuated vaccine [15]. BHK-21 cells were used to rescue virus by transfection with *in vitro*-transcribed RNA. Marc-145 cells were used for virus rescue and subsequent experiments. Cells were maintained as previously described [10].

### RNA extraction and molecular cloning of viral genomic cDNA fragments

**Figure 1:**
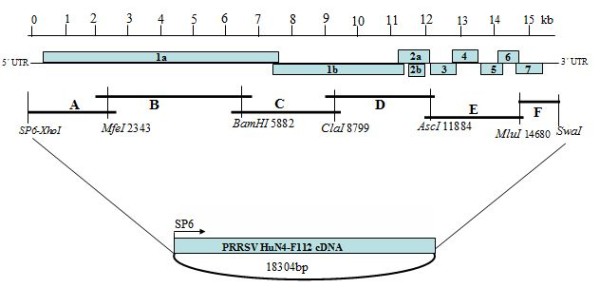
**Strategy for the construction of the full-length cDNA clone of an attenuated live PRRSV vaccine strain, HuN4-F112**. The organization of the viral genome is shown, as are the positions of the unique restriction sites used for cloning purposes. The numbers 1a, 1b, 2a, 2b and 3 through 7 indicate the PRRSV open reading frames. An SP6 RNA promoter with two nontemplated G residues preceded the viral genome. The complete viral genome was divided into six fragments flanked by unique restriction sites, represented by the horizontal lines labeled A through F. The fragments were inserted into the modified pBluescript II SK(+) vector.

Total RNAs were isolated from the supernatants of MARC-145 cells culture at 48 h post infection (PI) by using a QIAamp viral RNA kit (QIAGEN). The cDNA synthesis was performed with SupersciptIII reverse transcriptase (Invitrogen) and reverse primers of each fragment (Table 1). The PCR primers in Table 1 were designed mainly according to the sequence of PRRSV HuN4 strain with the GeneBank accession number of EF635006[10]. Six fragments (designated as A, B, C, D, E and F. Figure 1). spanning the full-length genome, were subsequently generated by PCR amplification with *Platinum^® ^Taq *DNA polymerase. Each PCR product was cloned into pCR-Blunt II-TOPO vector (Invitrogen). Nucleotide sequencing of the clones was performed by Shanghai Auke Inc. (Shanhai, China) using an ABI 377 automatic sequencer. The sequence data were analyzed using the Lasergene Software Package (DNAstar Inc., USA).

**Table 1:** Primers used for amplification of the genome of PRRSV HuN4-F112.

Primer^a^	Sequences^b ^(5'-3')	Position in HuN4
For fragment A		
F16(sp6)	CCGCTCGAGTTAATTAA*ATTTAGGTGACACTATA***GG**ATGACGTATAGGTGTT	1-16
R2355	GTGATGAACCTCGTCACCTTGTGCAGGG	2355-2382
For fragment B		
F2300	CTTTGGGCAAGGACTCGGT	2300-2319
R5914	GATCCTGTGTGAACGCCGAC	5914-5933
For fragment C		
F5853	CTTCTGCTTCACCGCGTGT	5853-5871
R8825	AAGAAGATTGGCGGCAAAC	8825-8843
For fragment D		
F8764	GCAGGTGCCTTGAAGCTGAT	8764-8783
R11910	CTCATGCTGATGGCATTAGC	11910-11929
For fragment E		
F11851	AGGACTGGGAGGATTACAAT	11851-11870
R14670	CGGACGACAAA**C**GCGTGGTTAT	14670-14691
For fragment F		
F14668	TGATAACCACGC**G**TTTGTCGTC	14668-14689
R15313	TATAGCGGCCGC*ATTTAAAT*(T)_32_AATTACGG	15313-15320

*^a ^*Primer names are organized in groups. Prefixes: F, forward PCR primer; R, reverse PCR primer.

^b ^The SP6 RNA polymerase promoter sequence in primer F16 is shown in italics. Restriction sites introduced by PCR are underlined and specified in parentheses at the end of the sequence. Silent mutations within the viral sequence are shown in boldface. Fragments A through F correspond to the letters in Fig. 1.

### Construction of full-length cDNA clone of HuN4-F112

The strategy for construction of full-length cDNA clone of HuN4-F112 is illustrated in Figure 1. A total of six fragments, covering the complete HuN4-F112 genome, were subsequently PCR amplified with *Platinum*^® ^*pfx *DNA polymerase according to the manufacturer's protocol. The A at position 14680 was mutated to G by PCR to create a restriction enzyme site *Mlu*I, a translationally silent substitution, both for fragment E and F ligation and as a genetic marker to differentiate the cloned virus and the parental virus. The bacteriophage SP6 RNA polymerase promoter (underlined sequence) and two non-viral guanosine residues were engineered into primer F16 (Table 1.).

The vector, pBluescript II SK(+) (Stratagene) was modified, the T7 RNA promoter in the vector was changed into the SP6 RNA promoter by PCR mutagenesis. The fragment between the *Xho*I and *Not*I sites were replaced by a stuffer fragment, which was prepared as a synthetic gene containing the restriction enzyme sites as shown in Figure 1. The PCR-amplified fragments were gel purified, digested with enzymes as indicated in Figure 1, and cloned into the modified pBluescript II SK(+) vector. After each ligation step, the constructed plasmid was transformed into *Escherichia coli *DH5α cells and grown overnight at 37°C in the presence of ampicillin. Over-lapped cDNA fragments were joined by using shared restriction sites (Figure 1). Finally, a full-length HuN4-F112 cDNA clone, pHuN4-F112, was obtained (Figure 1). The completely assembled full-length cDNA clone was sequenced.

### In vitro transcription and transfection

The full-length cDNA clone was linearized by cleavage with restriction enzyme *Swa*I, which cuts downstream of the poly (A) tail. Linearized plasmid DNA was used for in vitro transcription of capped RNA with the mMessage High Yield Capped RNA Transcription kit (Ambion) according to the manufacturer's instructions and including treatment of the RNA with DNase to remove input plasmid. The transcribed RNA was purified with a MEGA clear kit (Ambion) following the manufacturer's instruction and quantified by spectrophotomery. The synthetic RNA was transfected into BHK-21 cells using DMRIE-C reagent (Invitrogen) according the protocol recommended by the manufacturer. To rescue the virus, cell culture supernatant obtained 24 h post-transfection was serially passaged on MARC-145 cells. Rescue of infectious virus was confirmed by indirect immunofluorescence assay (IFA) using anti-N monoclonal antibody N3H2 [16].

### Discrimination between the cloned virus and the parental virus

Viral RNAs were extracted from cell culture supernatants of the infected cells by using an RNeasy Plus Mini kit (QIAGEN). RT-PCR was performed with the pairs of primer: JD1, 5'-CACAGCTCCACAGAAGGTGC-3' and JD2, 5'-TAACAGCTTTTCTGCCACCC-3'. The amplified product was digested by *Mlu*I to check the genetic marker engineered into the cloned virus.

### In vitro stability and growth kinetics of the rescued virus

To assess the replication stability of the rescued viruses, the cloned viruses were serially passaged (F4 to F6) in MARC-145 cells, and the CPE and the presence of genetic markers in the viruses were observed. Virus titers in cell cultures for each of passage were determined by a microtitration infectivity assay and recorded as TCID_50 _per milliliter by using the Reed-Muench method. Briefly, cells were prepared in 96-well plates and inoculated with virus suspensions (100 μL/well), which were prepared by serial 10-fold dilution. After absorption for 1 h at 37°C, the liquids in the wells were removed, and DMEM with 5% FBS was added to the wells. Plates were incubated for an additional 72 to 96 h; virus titers were determined by the presence of a visible CPE.

Growth kinetics studies were performed by infecting MARC-145 cells with the rescued virus and parental virus at an MOI of 0.1. After 1 h of virus adsorption, cells were washed and incubated in DMEM with 3% FBS at 37°C. The virus-infected supernatants were collected at various time points and viral titers were determined and expressed as TCID_50 _per milliliter.

## Results

### Sequence determination of the PRRSV HuN4-F112 genome

The PRRSV HuN4-F112 strain genome was determined to be 15352 nucleotides in length. The genome contain nine deduced open reading frames (ORF) which is flanked by a 5'untranslated region (5'UTR) of 189 nt and a 3'untranslated region (3'UTR) of 150 nt excluding the poly(A) tail, respectively.

### Construction of full-length cDNA clone of HuN4-F112 and determination of its infectivity

**Figure 2:**
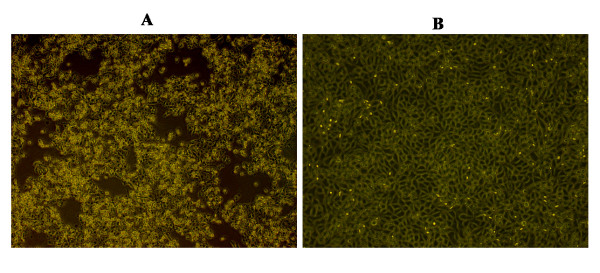
**Cytopathic effect (CPE) of the cloned virus in Marc-145 cells**. Supernatants from the transfected BHK-21 cells obtained 24 h post-transfection were serially passaged on Marc-145 cells, at 72 h post-infection (p.i.), CPE was observed in rescued virus (A), while mock-infected cells remained normal (B).

**Figure 3:**
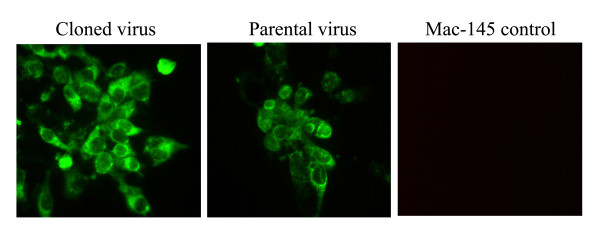
**Immunofluorescence assay**. MARC-145 cells infected with the 3rd passage culture of the viruses were fixed 48 h post-inoculation and examined by immunofluoresence assay with the anti-N monoclonal antibody (N3H2) of PRRSV.

The plasmid pHuN4-F112 that contained the full-length cDNA of the entire viral genome of the HuN4-F112 was linearized by digestion with *Swa*I, and used for in vitro transcription by SP6 RNA polymerase to synthesize capped RNAs. To recover infectious virus from the full-length cDNA clone, BHK-21 cells were transfected with the capped RNA with the transfection reagent DMRIE-C. Supernatants from the transfected BHK-21 cells obtained 24 h post transfection were serially passaged on Marc-145 cells, at 72 h post-infection (p.i.), cytopathic effects (CPEs) was observed in cells (Figure 2). Three days following transfection of MARC-145 cells with supernatants of cells infected with cloned and parental virus, immunofluorescence assay (IFA) was performed to examine the expression of nucleocapsid (N) protein. As shown in Figure 3, the N proteins of both cloned and parental virus were expressed in MARC-145 cells. Thus, our results show that infectious virus was rescued from BHK-21 and MARC-145 cells transfected with capped in vitro-transcribed RNA.

### Discrimination between cloned virus and parental virus

**Figure 4:**
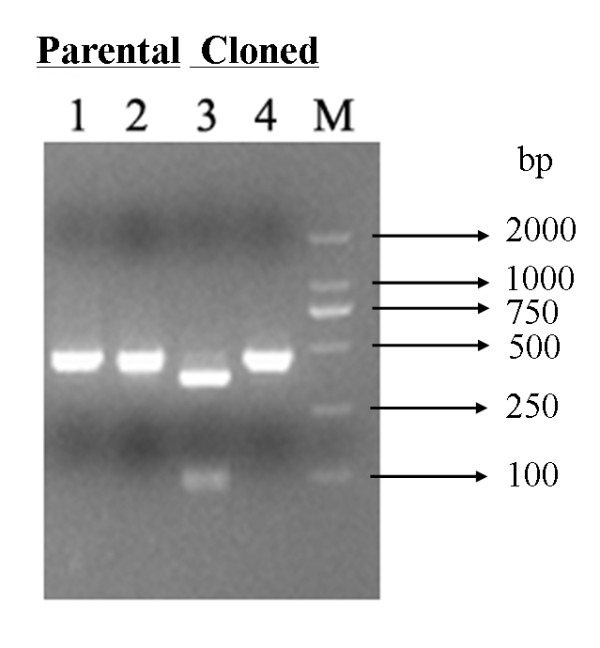
**Differentiation between cloned virus and parental HuN4-F112 strain**. A *Mlu*I restriction site was introduced in the full-length cDNA clone of HuN4-F112 to allow discrimination between cloned virus (tagged with the *Mlu*I site) and parental virus (lacks the *Mlu*I site). The presence of a *Mlu*I restriction site resulted in fragments of 460 bp and 92 bp. 1 and 4 were RT-PCR fragments undigested, 2 and 3 were RT-PCR fragments digested by *Mlu*I.

To differentiate the rescued viruses and parental viruses, a genetic marker, *Mlu*I site at nt 14680, was engineered into the genome length cDNA. As expected, the RT-PCR fragment derived from the cloned virus was cleaved by *Mlu*I, generating two fragments of 360 bp and 92 bp (Figure 4). In contrast, the PCR amplification products derived from the parental isolate was not cleaved by *Mlu*I (Figure 4).

### Characterization of the cloned virus

**Figure 5:**
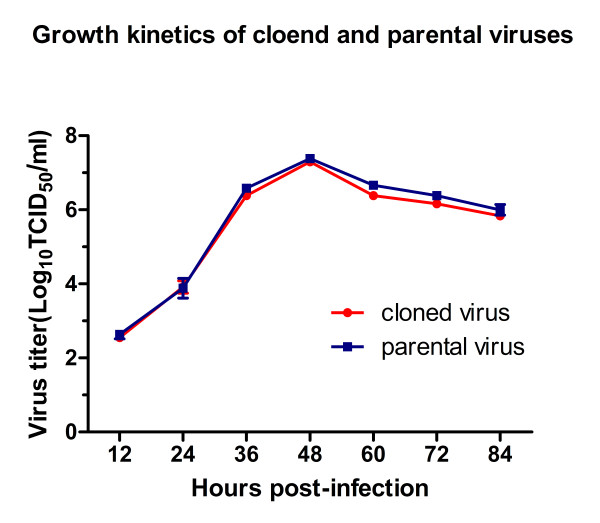
**Growth kinetics of cloned and parental virus**. The growth curves of the rescued virus and parental virus were drawn by assaying the viral titers of the supernatants obtained from 12 h to 84 h postinfection by using microtitration infectivity assays. Data are calculated and shown as means ± standard deviations (error bars) from three independent trials.

Growth kinetics studies were performed to compare the growth of rescued virus and parental virus in MARC-145 cells. Results (Figure 5) show that growth kinetics of rescued virus and parental virus was not significantly different from each other and that titers peaked at 48 h pi for both viruses. The result indicates that the rescued virus possesses growth characteristics similar to the parental virus.

## Discussion and conclusions

In this study, we described the complete nucleotide sequence of the viral genome and the construction of infectious cDNA clone of HuN4-F112, a live-attenuated North-American-type PRRSV vaccine strain. Transfection of capped in vitro transcripts, derived from this clone, into BHK-21 cells and Marc-145 cells led to efficient recovery of infectious virus from cultured cells. Indeed, at 3 days post-transfection, CPE was observed in transfected cells. The rescued virus displayed similar growth properties to its parent virus in MARC-145 cells. Detection of the *Mlu*I genetic marker indicates that the rescued virus remained genetically stable during passage in cells.

Since May 2006, atypical PRRS (so-called porcine high fever syndrome (PHFS) in china) was pandemic in china. Several studies have confirmed that the causative agent of this outbreak was highly pathogenic PRRSV (HP-PRRSV), several HP-PRRSV strains were isolated and their reverse genetic systems were developed by different labs [13,17-20]. We also isolated an atypical PRRS strain named HuN4 and attenuated this strain in vitro by passaging on Marc-145 cells, the attenuated strain was designated HuN4-F112 (112^th ^passage). Study shows that HuN4-F112 was sufficiently attenuated and antigenic enough to confer protection against a lethal wild-type challenge [10,15].

Sequence alignment between HuN4-F112 and its parental virulent isolate HuN4 showed that both the 5'UTR and 3'UTR are identical and mutations are presented in both nonstructural and structural protein regions. We surmise that the basis of PRRSV attenuation may be multifactor. A recent study also confirmed this by constructing of a series of chimeric viruses where specific genomic regions of a highly virulent PRRSV infectious clone (FL12) were replaced with their counterparts of an attenuated vaccine strain PrimePac. Results indicate that NSP3-8 and GP5 are the location of major virulence determinants, while other virulence determinants may also be contained in NSP1-3, NSP10-12 and GP2 [21]. The availability of the complete genome sequence and infectious clone of the virulence-attenuated strain HuN4-F112 would make it possible to use a comparative virology approach to dissect the virulence determinants of PRRSV. This approach will likely yield new information on PRRSV virulence regulation and virus-host interaction in the future.

In conclusion, we rescued virus from an infectious cDNA clone of attenuated vaccine. It is possible in the future that a new attenuated PRRSV vaccine with broader specificity and good immunogenicity can be designed in vitro via an infectious cDNA clone platform coupled with validated information on virulence determinants.

## Competing interests

The authors declare that they have no competing interests.

## Authors' contributions

GT and YZ have been involved in conception and design of the study, and revising the manuscript critically; SZ has been involved in design of this study, performed the study and collected data, and prepared the first draft of the manuscript; YF participated in design of the study. GL, LY and HY participated in data analysis. All authors read and approved the final manuscript.
